# A case of napsin A-positive metastatic lung cancer originating from the colon

**DOI:** 10.1186/s40792-017-0389-9

**Published:** 2017-11-09

**Authors:** Tomokazu Ito, Kozo Nakanishi, Hidenori Goto

**Affiliations:** Department of General Thoracic Surgery, National Hospital Organization, Saitama National Hospital, 2-1 Suwa, Wako, Saitama, 351-0102 Japan

**Keywords:** Immunochemical stain, Colon cancer, Lung cancer

## Abstract

**Background:**

We report a case of napsin A-positive metastatic lung cancer originating from the colon. No cases of napsin A-positive metastatic lung tumors originating from colorectal cancer have been reported previously.

**Case presentation:**

Computed tomography identified a small lung nodule in a 70-year-old male patient, 18 months after resection for rectal cancer. The size of the lung tumor increased from 1.8 to 2.1 cm in 6 months and metastasis from the rectal cancer was suspected. Resection of the lung tumor was performed, and the histological features of the lung tumor revealed findings typical of colorectal adenocarcinoma and resembled those of the original rectal cancer. Furthermore, the metastasis stained positive for napsin A and thyroid transcription factor-1 (TTF-1) on immunohistochemical evaluation, and immunohistochemical analysis identified the same results in the rectal specimen.

**Conclusions:**

These findings led us to believe that this was a rare case of napsin A-positive metastatic lung cancer originating in the colon. The patient was treated with chemotherapy for recurrent rectal cancer, and no other metastases were found after the lung resection. This is the first report of napsin A-positive colorectal cancer metastasizing to the lung.

## Background

Napsin A and thyroid transcription factor-1 (TTF-1) are known to be useful immunohistochemical markers to distinguish primary lung cancers from metastatic lung tumors. These markers are usually positive in primary lung adenocarcinomas but not metastatic adenocarcinomas in other organs. Napsin A, especially, is rarely expressed in colon adenocarcinomas and has high specificity in lung adenocarcinoma. No cases of Napsin A-positive metastatic lung tumors originating from colorectal cancer have been reported previously.

We report a patient with a metastatic lung tumor originating from rectal cancer that was positive for both napsin A and TTF-1 expression.

## Case presentation

**Fig. 1:**
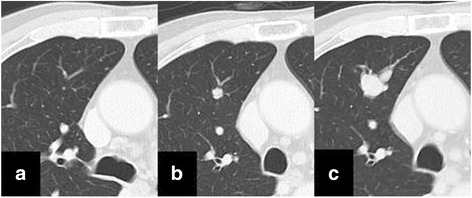
Chest computed tomography. There was no tumor shadow in the lung when the resection for rectal cancer was performed (**a**). A solid nodule in the right upper lung lobe was first detected 18 months after surgery (**b**). The nodule grows in diameter 6 months later (**c**)

A 70-year-old man underwent colorectal resection for rectal cancer (well-differentiated tubular adenocarcinoma, pT3a N0, stage IIA). At this time, no pulmonary lesion was detected on computed tomography (CT) (Fig. 1a). Eighteen months later, a well-defined solid nodule appeared in the upper lobe of the right lung on CT (Fig. 1b). The diameter of the nodule was 0.8 cm. After a further 6 months, the diameter increased up to 2.1 cm (Fig. 1c). Positron emission tomography revealed high uptake in the nodule. Serum levels of carcinoembryonic antigen and CA19-9 were within the normal range, and no recurrence of the rectal cancer was found.

Based on radiological findings and the speed of growth, the lung nodule was clinically diagnosed as a metastatic tumor originating from the rectal cancer. Thoracoscopic wedge resection of the right upper lung lobe was subsequently performed. A frozen section of the resected tumor was examined perioperatively. The intraoperative pathological diagnosis was metastatic adenocarcinoma, and the surgery was completed without additional resection. The postoperative course was uneventful, and the patient was discharged on the fourth postoperative day.

**Fig. 2:**
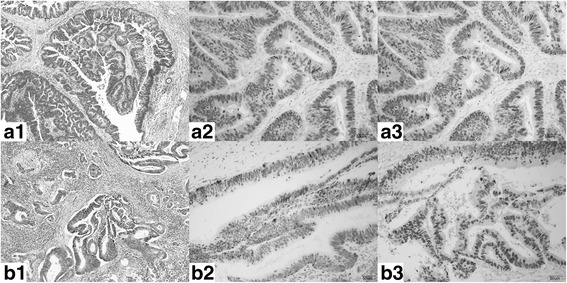
Histological findings and immunohistochemical studies. a Specimens of the lung tumor. b Specimens of the rectal lesion; 1. hematoxylin-eosin stain; 2. Napsin A expression (monoclonal antibody IP64); 3. TTF-1 expression (monoclonal antibody SPT24). Hypercolumnar, atypical, papillary-tubular cells proliferated and were histologically diagnosed as adenocarcinoma (**a1**, **b1**). Of both the lung specimens and the rectal specimen, some tumor cells contained regions that were be positive for napsin A regardless of the staining method (**a2**, **b2**). Almost all the tumor cells of both specimens were positive for TTF-1 (**a3**, **b3**)

Further pathological examination of the surgical specimens including the previously resected rectal cancer was performed. The histological features of the lung tumor revealed findings typical of colorectal adenocarcinoma (Fig. 2a1) and resembled those of the original rectal cancer (Fig. 2b1). Furthermore, immunohistochemical study of the lung tumor was performed with multiplex antibody reagent (Pathology Institute corporation, Toyama, Japan) containing napsin A (clone: TMU-Ad02) and TTF-1 (clone: SPT24). The study showed that both markers were positive. The specimens were reexamined with polyclonal napsin A antibodies (Nichirei Biosciences Inc., Tokyo, Japan) and the rabbit monoclonal TTF-1 antibody (clone: SP141, Roche) in separate experiments. In addition, we obtained an immunohistochemical stain from a third-party laboratory (Kyodo Byori Inc., Kobe, Japan). They used two monoclonal antibodies; napsin A (clone: IP64, Leica Biosystems Newcastle Ltd.) (Fig. 2a2, b2); TTF-1 (clone: SPT24, Novocastra) (Fig. 2a3, b3). All results were positive for both markers. Thus, we finally diagnosed the lung nodule as a metastasis from the napsin A-positive rectal cancer.

The patient then received eight cycles of combined chemotherapy with oxaliplatin and capecitabine. The patient is alive without any recurrence 34 months after the first surgery for primary rectal cancer.

## Discussion

To our knowledge, this is the first report of a napsin A-positive metastatic lung tumor originating from colon cancer. Napsin A is generally expressed in lamellar bodies of type II pneumocytes [1]. Therefore, napsin A is highly expressed in primary lung adenocarcinomas, but not metastatic lung adenocarcinomas [2]. The specificity of napsin A expression is reliable especially as compared with colon adenocarcinomas. Renal cell carcinomas and a few other adenocarcinomas are known to be occasionally positive for napsin A; however, no positive colon adenocarcinoma cases have been reported [3].

It has been reported that polyclonal napsin A antibody sometimes reacts nonspecifically to non-pulmonary adenocarcinomas [4]. In our case, immunohistochemical examination was performed with various antibodies: cocktailed, polyclonal, and monoclonal. Reexamination was performed at a third-party laboratory, and all the results were positive in the both the lung tumor and rectal cancer.

The diagnosis in our case was metastatic lung cancer originating from primary rectal cancer. The histological features of the lung tumor were typical of a primary rectal adenocarcinoma. The radiological features of the lung tumor were also typical of a metastatic lung tumor. Considering the clinical course of the patient, it is irrational to believe that a primary lung cancer appeared 18 months after resection of a metastatic rectal cancer.

The lung cancer and the rectal cancer could not have been independent of each other. The results of a hematoxylin and eosin stain were similar in both lesions, as was the protein expression pattern; as such, both adenocarcinomas likely share a common origin. No other malignant lesions have been found 21 months after the last surgery.

In this patient, the colon adenocarcinoma was positive for napsin A. Some thyroid carcinomas have also been reported to be positive for napsin A, which were all of the papillary type with tall cell morphology [3]. Our patient’s rectal cancer also exhibited these features. The reasons for positive staining of napsin A in our case remain unknown. The rectal cancer may have produced some proteins that cross-react to napsin A [5] or there may be issues with the sensitivity of the antibodies used for immunohistochemical analysis.

One case of TTF-1-positive metastatic lung cancer originating from colon cancer has already been reported [6]. The expression of TTF-1 in primary lung adenocarcinomas is high but lower than napsin A. TTF-1 expression is rarely positive in cancers of the colon, uterus, ovaries, and so on [7].

## Conclusions

We report a case of napsin A- and TTF-1-positive metastatic lung cancer originating from rectal cancer. Clinicians and pathologists should be aware of the existence of false-positive cases like this and be extra vigilant in similar cases in the future.

## Abbreviations

CT: Computed tomography
TTF-1: Thyroid transcription factor-1
